# Zika-Associated Birth Defects Reported in Pregnancies with Laboratory Evidence of Confirmed or Possible Zika Virus Infection — U.S. Zika Pregnancy and Infant Registry, December 1, 2015–March 31, 2018

**DOI:** 10.15585/mmwr.mm7103a1

**Published:** 2022-01-21

**Authors:** Nicole M. Roth, Megan R. Reynolds, Elizabeth L. Lewis, Kate R. Woodworth, Shana Godfred-Cato, Augustina Delaney, Amanda Akosa, Miguel Valencia-Prado, Maura Lash, Amanda Elmore, Peter Langlois, Salma Khuwaja, Aifili Tufa, Esther M. Ellis, Eirini Nestoridi, Caleb Lyu, Nicole D Longcore, Monika Piccardi, Leah Lind, Sharon Starr, Loletha Johnson, Shea E. Browne, Michael Gosciminski, Paz E. Velasco, Fern Johnson-Clarke, Autumn Locklear, Mary Chan, Jane Fornoff, Karrie-Ann E. Toews, Julius Tonzel, Natalie S. Marzec, Shelby Hale, Amy E. Nance, Teri Willabus, Dianna Contreras, Sowmya N. Adibhatla, Lisa Iguchi, Emily Potts, Elizabeth Schiffman, Katherine Lolley, Brandi Stricklin, Elizabeth Ludwig, Helentina Garstang, Meghan Marx, Emily Ferrell, Camille Moreno-Gorrin, Kimberly Signs, Paul Romitti, Vinita Leedom, Brennan Martin, Louisa Castrodale, Amie Cook, Carolyn Fredette, Lindsay Denson, Laura Cronquist, John F. Nahabedian, Neha Shinde, Kara Polen, Suzanne M. Gilboa, Stacey W. Martin, Janet D. Cragan, Dana Meaney-Delman, Margaret A. Honein, Van T. Tong, Cynthia A. Moore

**Affiliations:** ^1^Division of Birth Defects and Infant Disorders, National Center on Birth Defects and Developmental Disabilities, CDC; ^2^Eagle Global Scientific, LLC, San Antonio, Texas; ^3^Puerto Rico Department of Health; ^4^New York City Department of Health and Mental Hygiene, New York; ^5^Florida Department of Health; ^6^Texas Department of State Health Services; ^7^Houston Health Department, Houston, Texas; ^8^American Samoa Department of Health; ^9^U.S. Virgin Islands Department of Health; ^10^Massachusetts Department of Public Health; ^11^Los Angeles County Department of Public Health, Los Angeles, California; ^12^New York State Department of Health; ^13^Maryland Department of Health; ^14^Pennsylvania Department of Health; ^15^Philadelphia Department of Public Health, Philadelphia, Pennsylvania; ^16^New Jersey Department of Health; ^17^Virginia Department of Health; ^18^Rhode Island Department of Health; ^19^Kosrae State Department of Health Services, Federated States of Micronesia; ^20^District of Columbia Department of Health, Washington, DC; ^21^North Carolina Department of Health and Human Services; ^22^Washington State Department of Health; ^23^Illinois Department of Public Health; ^24^Chicago Department of Public Health, Chicago, Illinois; ^25^Louisiana Department of Health; ^26^Colorado Department of Public Health and Environment; ^27^Ohio Department of Health; ^28^Utah Department of Health; ^29^Georgia Department of Public Health; ^30^Arizona Department of Health Services; ^31^Wisconsin Department of Health Services; ^32^Oregon Health Authority; ^33^Indiana Department of Health; ^34^Minnesota Department of Health; ^35^Tennessee Department of Health; ^36^Arkansas Department of Health; ^37^Nebraska Department of Health and Human Services; ^38^Republic of the Marshall Islands Ministry of Health and Human Services; ^39^South Dakota Department of Health; ^40^Kentucky Department of Public Health; ^41^Delaware Health and Social Services; ^42^Michigan Department of Health and Human Services; ^43^University of Iowa College of Public Health, Iowa City, Iowa; ^44^South Carolina Department of Health and Environmental Control; ^45^Vermont Department of Health; ^46^Alaska Department of Health and Social Services; ^47^Kansas Department of Health and Environment; ^48^New Hampshire Department of Health and Human Services; ^49^Oklahoma State Department of Health; ^50^North Dakota Department of Health; ^51^Division of Vector-Borne Diseases, National Center for Emerging and Zoonotic Infectious Diseases, CDC.

Zika virus infection during pregnancy can cause serious birth defects of the brain and eyes, including intracranial calcifications, cerebral or cortical atrophy, chorioretinal abnormalities, and optic nerve abnormalities (*1**,**2*). The frequency of these Zika-associated brain and eye defects, based on data from the U.S. Zika Pregnancy and Infant Registry (USZPIR), has been previously reported in aggregate (*3*,*4*). This report describes the frequency of individual Zika-associated brain and eye defects among infants from pregnancies with laboratory evidence of confirmed or possible Zika virus infection. Among 6,799 live-born infants in USZPIR born during December 1, 2015–March 31, 2018, 4.6% had any Zika-associated birth defect; in a subgroup of pregnancies with a positive nucleic acid amplification test (NAAT) for Zika virus infection, the percentage was 6.1% of live-born infants. The brain and eye defects most frequently reported included microcephaly, corpus callosum abnormalities, intracranial calcification, abnormal cortical gyral patterns, ventriculomegaly, cerebral or cortical atrophy, chorioretinal abnormalities, and optic nerve abnormalities. Among infants with any Zika-associated birth defect, one third had more than one defect reported. Certain brain and eye defects in an infant might prompt suspicion of prenatal Zika virus infection. These findings can help target surveillance efforts to the most common brain and eye defects associated with Zika virus infection during pregnancy should a Zika virus outbreak reemerge, and might provide a signal to the reemergence of Zika virus, particularly in geographic regions without ongoing comprehensive Zika virus surveillance.

To monitor the impact of the 2015–2017 Zika virus outbreak, CDC, in collaboration with state, local, and territorial health departments, established USZPIR to conduct mother-infant linked longitudinal surveillance of outcomes in pregnant women and infants with laboratory evidence of confirmed or possible Zika virus infection during pregnancy* in the 50 U.S. states, the District of Columbia (DC), U.S. territories, and freely associated states.^†^ Data from this cohort have been published previously (*3*–*5*). Pregnancies with an outcome occurring during December 1, 2015–March 31, 2018, were included in USZPIR with data reported as of December 2020 included in this report. Jurisdictions collected prenatal, pregnancy outcome, and follow-up information for infants and children (from birth through age 5 years)^§^ from medical records in a standardized format. 

All mother-infant data with an indication of a possible abnormality were reviewed by subject matter experts (which included CDC clinicians and researchers and external consultants); data reviewed included results from neuroimaging, ophthalmologic examinations, and clinical examinations for any criteria based on USZPIR surveillance case definition (*6*). Cases that met criteria for Zika-associated abnormalities were subsequently reviewed in detail by two or more clinicians (including pediatricians, obstetrician-gynecologists, and clinical geneticists), for confirmation and classification of the individual defect or defects. All discrepancies in classification were discussed and resolved among a panel of experts. Infants who had microcephaly and were not small for gestational age at birth underwent further review; those who met criteria for a potential birth head circumference measurement inaccuracy were not included as having microcephaly in USZPIR.^¶^ Infants with other abnormal radiographic findings (e.g., mineralizing vasculopathy, and isolated subependymal cysts), which were deemed as having “unknown clinical significance” by experts, were not reported.

In this report, the number of infants with any Zika-associated birth defect and enumerated individual brain and eye defects identified in the entire cohort with laboratory evidence of confirmed or possible Zika virus infection from a maternal, placental, fetal, or infant specimen are presented. A subgroup of infants from pregnancies with confirmed Zika virus infection (i.e., positive Zika virus NAAT) are reported to examine whether findings are consistent with the entire cohort.** Zika-associated birth defects among pregnancy losses are reported separately.^††^ In addition, the frequency of Zika-associated birth defects by location of birth, trimester with first evidence of Zika virus exposure (based on symptom onset, travel history to a region with endemic Zika virus transmission, or positive laboratory results), maternal symptom status, and reported neuroimaging and ophthalmology examinations are presented. Analyses were conducted using SAS (version 9.4; SAS Institute). CIs were calculated using exact Poisson regression. This activity was reviewed by CDC and was conducted consistent with applicable federal law and CDC policy.^§§^

During December 1, 2015–March 31, 2018, among 6,799 live-born infants reported in USZPIR, 2,288 (33.7%) were born in U.S. states and DC and 4,511 (66.3%) in U.S. territories and freely associated states (Table 1). Zika virus exposure was reported for 2,121 (31.2%) pregnant women in the first trimester; 2,495 (36.7%) in the second trimester; and 2,039 (30.0%) in the third trimester. Symptoms compatible with Zika virus disease^¶¶^ were reported in 35% of these women.

**TABLE 1:** Frequency of Zika-associated birth defects,* by selected characteristics among live-born infants from pregnancies with laboratory evidence of confirmed or possible Zika virus infection — U.S. Zika Pregnancy and Infant Registry, December 1, 2015–March 31, 2018

Characteristic	From pregnancies with laboratory evidence of confirmed or possible Zika virus infection^†^		From pregnancies with positive Zika virus NAAT result^§^	
	No./Total no.	% (95% CI)	No./Total no.	% (95% CI)
**Total**	**315/6,799**	**4.6 (4.1–5.2)**	**138/2,257**	**6.1 (5.1–7.2)**
**Location of birth**				
U.S. states and DC	124/2,288	5.4 (4.5–6.5)	38/374	10.2 (7.2–14.0)
U.S. territories and freely associated states**^¶^**	191/4,511	4.2 (3.7–4.9)	100/1,883	5.3 (4.3–6.5)
Trimester with first evidence of exposure**,^††^				
1st**^§§^**	108/2,121	5.1 (4.2–6.2)	43/539	8.0 (5.8–10.8)
2nd	107/2,495	4.3 (3.5–5.2)	62/1,028	6.0 (4.6–7.7)
3rd	82/2,039	4.0 (3.2–5.0)	25/657	3.8 (2.5–5.6)
Maternal symptoms^¶¶,^***				
Signs/Symptoms of Zika virus disease	126/2,379	5.3 (4.4–6.3)	92/1,596	5.8 (4.7–7.1)
No signs/symptoms of Zika virus disease	186/4,382	4.2 (3.7–4.9)	46/661	7.0 (5.1–9.3)
**Examinations reported**				
Neuroimaging	258/4,086	6.3 (5.6–7.1)	120/1,595	7.5 (6.2–9.0)
Ophthalmology	167/2,456	6.8 (5.8–7.9)	79/1,072	7.4 (5.8–9.2)

**Abbreviations:** DC = District of Columbia; NAAT = nucleic acid amplification test; RT-PCR = reverse transcription–polymerase chain reaction; USZPIR = U.S. Zika Pregnancy and Infant Registry.

* Zika-associated birth defects include selected congenital brain anomalies (intracranial calcifications, cerebral or cortical atrophy, abnormal cortical gyral patterns, corpus callosum abnormalities, cerebellar abnormalities, porencephaly, hydranencephaly, or ventriculomegaly/hydrocephaly); selected congenital eye anomalies (microphthalmia or anophthalmia; coloboma; cataract; intraocular calcifications; chorioretinal anomalies involving the macula, excluding retinopathy of prematurity; and optic nerve atrophy, pallor, and other optic nerve abnormalities); and/or microcephaly at birth (birth head circumference below the third percentile for infant sex and gestational age based on INTERGROWTH-21st online percentile calculator unless infants meet criteria of possible measurement inaccuracy. http://intergrowth21.ndog.ox.ac.uk/

^†^ Includes maternal, placental, or infant laboratory evidence of confirmed or possible Zika virus infection during pregnancy based on presence of Zika virus RNA by a positive NAAT (e.g., RT-PCR), serologic evidence of a Zika virus infection, or serologic evidence of an unspecified flavivirus infection.

^§^ Includes maternal, placental, or infant laboratory evidence of confirmed Zika virus infection during pregnancy based on presence of Zika virus RNA by a positive NAAT (e.g., RT-PCR).

^¶^ U.S. territories in USZPIR are American Samoa, Puerto Rico, and the U.S. Virgin Islands; freely associated states are Federated States of Micronesia and the Marshall Islands.

** Among pregnancies in which birth occurred in the U.S. states and DC, symptom onset date, travel dates to an endemic region, or date of earliest laboratory evidence of Zika virus infection were used to calculate trimester of exposure. Among pregnancies in which birth occurred in U.S. territories and freely associated states, symptom onset date or date of earliest laboratory evidence of Zika virus infection were used to calculate trimester of exposure.

^††^ Unknown trimester of exposure is not shown because of small cell sizes; 144 pregnancies were missing trimester of exposure.

^§§^ Zika virus infections that occurred during the periconceptual period, which is defined as 4 weeks before last menstrual period, are included in the first trimester of exposure.

^¶¶^ Maternal symptom status is not shown because of small cell sizes; 38 pregnancies were missing maternal symptom status.

*** Signs and symptoms included fever, arthralgia, conjunctivitis, rash, and other clinical signs or symptoms that are consistent with Zika virus disease.

Among live-born infants reported in USZPIR, 4.6% (315 of 6,799) had any Zika-associated birth defect. In the subgroup with positive Zika virus NAAT during pregnancy, 6.1% (138 of 2,257) infants had any Zika-associated birth defect. Among pregnancies with positive Zika virus NAAT results, and thus less likelihood of exposure misclassification, the frequency of any Zika-associated birth defect was higher among those with first*** (8.0%) and second (6.0%) trimester infections compared with third trimester infections (3.8%). Frequency of Zika-associated birth defects in infants was similar among those born to symptomatic (5.3%) and asymptomatic (4.2%) pregnant women; neuroimaging and ophthalmology examinations were reported for 4,086 (60.1%) and 2,456 (36.1%), respectively.

The most frequent structural defects reported among live-born infants and children were microcephaly; corpus callosum abnormalities; intracranial calcification; abnormal cortical gyral patterns; ventriculomegaly; cerebral or cortical atrophy; chorioretinal atrophy, scarring, or pigmentary changes; and optic nerve abnormalities (Table 2). A similar distribution of birth defects was observed in the total cohort and in the Zika virus NAAT-positive subgroup. Among infants with any Zika-associated birth defect, one third (110 of 315) had more than one birth defect identified.

**TABLE 2:** Individual Zika-associated birth defects among live-born infants from pregnancies with laboratory evidence of confirmed or possible Zika virus infection — U.S. Zika Pregnancy and Infant Registry, December 1, 2015–March 31, 2018

Birth defect	No. of infants (%)	
	From pregnancies with laboratory evidence of confirmed or possible Zika virus infection* (n = 6,799)	From pregnancies with positive Zika virus NAAT result^† ^(n = 2,257)
**Any Zika-associated birth defect^§^**	**315 (4.6)**	**138 (6.1)**
**Brain abnormalities/Microcephaly^¶^**		
Any brain abnormality/microcephaly	275 (4.0)	126 (5.6)
Microcephaly^**, ††^	214 (3.1)	100 (4.4)
Corpus callosum abnormalities	64 (0.9)	40 (1.8)
Intracranial calcifications	58 (0.9)	27 (1.2)
Abnormal cortical gyral patterns	56 (0.8)	29 (1.3)
Ventriculomegaly/Hydrocephaly	53 (0.8)	34 (1.5)
Cerebral or cortical atrophy	43 (0.6)	24 (1.1)
Cerebellar abnormalities	27 (0.4)	15 (0.7)
Fetal brain disruption sequence	12 (0.2)	10 (0.4)
Brainstem abnormalities	8 (0.1)	6 (0.3)
Porencephaly/Hydranencephaly	5 (0.1)	3 (0.1)
**Eye abnormalities**		
Any eye abnormality	76 (1.1)	34 (1.5)
Chorioretinal atrophy, scarring, or pigmentary changes	47 (0.7)	25 (1.1)
Optic nerve abnormalities	34 (0.5)	13 (0.6)
Coloboma	7 (0.1)	5 (0.2)
Congenital cataract	7 (0.1)	3 (0.1)
Microphthalmia	5 (0.1)	1 (—)
**Other brain and eye abnormality patterns**		
Multiple brain or eye abnormalities	110 (1.6)	55 (2.4)
Brain and eye abnormalities	36 (0.5)	22 (1.0)
One or more brain abnormalities only	239 (3.5)	104 (4.6)
One brain abnormality or microcephaly only	173 (2.5)	72 (3.2)
Microcephaly only^§§^	144 (2.1)	58 (2.6)
Microcephaly only and SGA	98 (1.4)	37 (1.6)
One or more eye abnormalities only	40 (0.6)	12 (0.5)
One eye abnormality only	32 (0.5)	11 (0.5)

**Abbreviations**: NAAT = nucleic acid amplification test; RT-PCR = reverse transcription–polymerase chain reaction; SGA = small for gestational age.

* Includes maternal, placental, or infant laboratory evidence of confirmed or possible Zika virus infection during pregnancy based on presence of Zika virus RNA by a positive NAAT (e.g., RT-PCR), serologic evidence of a Zika virus infection, or serologic evidence of an unspecified flavivirus infection.

^†^ Includes maternal, placental, or infant laboratory evidence of confirmed Zika virus infection during pregnancy based on presence of Zika virus RNA by a positive NAAT.

^§^ Zika-associated birth defects include selected congenital brain anomalies (intracranial calcifications, cerebral or cortical atrophy, abnormal cortical gyral patterns, corpus callosum abnormalities, cerebellar abnormalities, porencephaly, hydranencephaly, or ventriculomegaly/hydrocephaly); selected congenital eye anomalies (microphthalmia or anophthalmia; coloboma; cataract; intraocular calcifications; chorioretinal anomalies involving the macula, excluding retinopathy of prematurity; and optic nerve atrophy, pallor, and other optic nerve abnormalities); and/or microcephaly at birth (birth head circumference below the third percentile for infant sex and gestational age based on INTERGROWTH-21st online percentile calculator unless infants meet criteria of possible measurement inaccuracy. http://intergrowth21.ndog.ox.ac.uk/

^¶^ Among infants with brain abnormalities, microcephaly, or both, 24 (0.4%) and 11 (0.5%) infants also had arthrogryposis among pregnancies with laboratory evidence of confirmed or possible Zika virus infection during pregnancy and NAAT-confirmed Zika virus infection, respectively.

** Infants with birth head circumference below the third percentile based on INTERGROWTH-21st. http://intergrowth21.ndog.ox.ac.uk/

^††^ Among infants with microcephaly, 141 and 64 also had a birthweight below the 10th percentile (SGA) among pregnancies with laboratory evidence of confirmed or possible Zika virus infection during pregnancy and NAAT-confirmed Zika virus infection, respectively.

^§§^ Neuroimaging was available for 66.0% and 29.2% of infants with microcephaly only from pregnancies with laboratory evidence of confirmed or possible Zika virus infection during pregnancy and NAAT-confirmed Zika virus infection, respectively.

Among 325 pregnancies with laboratory evidence of confirmed or possible Zika virus infection that resulted in a pregnancy loss, 13 (4.0%) fetuses had any reported Zika-associated birth defect. Defects included microcephaly, cerebral or cortical atrophy, abnormal cortical gyral patterns, corpus callosum abnormalities, cerebellar abnormalities, hydranencephaly, ventriculomegaly or hydrocephaly, and brainstem abnormalities (C Moore, CDC, unpublished data, 2022). 

## Discussion

During 2015–2017, large Zika virus outbreaks occurred throughout the United States (including U.S. territories and freely associated states). In the United States, infections during pregnancy were initially reported among U.S. travelers returning from affected countries.^†††^ During 2016, widespread local transmission was documented in the territories of Puerto Rico and the U.S. Virgin Islands, and limited transmission was documented in some counties in Florida and Texas.^§§§^ Among completed pregnancies with laboratory evidence of Zika virus infection reported to USZPIR, 4.6% of live-born infants had any Zika-associated birth defect. Among the subgroup with NAAT-positive results, Zika-associated birth defects were reported with exposures throughout pregnancy but were more prevalent among infants born to mothers with exposure early in pregnancy. Approximately two thirds of pregnant women in this cohort reported asymptomatic infections.^¶¶¶^ The similar frequency of Zika-associated birth defects among asymptomatic and symptomatic pregnant women is consistent with previous findings (*3**,**5*).

Certain individual brain and eye defects associated with Zika virus infection were frequently reported in USZPIR cohort. A similar subset of Zika-associated birth defects was found to have significantly higher prevalence ratios in areas of widespread local transmission compared with areas without local transmission in the Zika Birth Defects Surveillance System.**** Given the short window for testing and that symptoms of Zika are often mild or absent, combining these two systems has identified the most prevalent Zika-associated birth defects. Using a surveillance system that monitored outcomes regardless of testing and a system that monitored outcomes among those possibly exposed to Zika virus has been critical to understanding the effects of Zika virus infection during pregnancy on infants and children.

The findings in this report are subject to at least five limitations. First, these data are based on information abstracted from medical records. Although CDC provided specific guidance for evaluation of all infants born from pregnancies with possible Zika virus exposure during pregnancy (*7*), these evaluations might not have been feasible, were not always conducted, or were not found in records (*4*). Zika-associated birth defects, especially individual brain and eye defects might not have been detected without occurrence and reporting of neuroimaging and ophthalmologic examinations. Second, these findings are only applicable to live births. Pregnancy losses are likely underreported to USZPIR, and among those reported, postnatal studies to verify prenatal findings or identify additional defects are often lacking. Third, although routine testing during pregnancy occurred in areas with local Zika virus transmission, a potential bias could have been introduced in areas without local transmission, as differential testing might have occurred in women reporting possible Zika virus exposure related to travel or sex or when birth defects were detected in the fetus or infant. Fourth, USZPIR surveillance case definition includes infants with microcephaly based on head circumference measurement at birth alone, and only one third of these had sufficient information to be evaluated for possible measurement error. Thus, misclassification of infants with microcephaly based on birth head circumference alone might still exist. Finally, pregnancies in persons with possible Zika virus exposure, including those with evidence of unspecified flavivirus infection were included; therefore, some might not have had Zika virus infection during pregnancy. Analysis of the subgroup with NAAT-positive results indicated higher frequency of any Zika-associated birth defects, but the distribution of individual defects was generally consistent between the total cohort and this subgroup.

Much has been learned since the first infant with Zika-associated birth defects was identified in the United States. This report is the first to describe Zika-associated birth defects from USZPIR with data combined from the U.S. states, DC, and U.S. territories and freely associated states. The study provides a description of the frequency of individual Zika-associated birth defects reported among infants from pregnancies with laboratory evidence of confirmed or possible Zika virus infection. Additional study is needed to define the full spectrum of Zika-associated outcomes, including any specific defects or combination of defects that might predict the presence of Zika virus infection and Zika virus circulation. Further monitoring of these infants for neurodevelopmental abnormalities is ongoing. Infants exposed to Zika virus infection in utero, but without structural birth defects, might also have neurologic sequelae and developmental delay (*4*,*8*). Zika virus outbreaks are tracked globally; Zika virus infection remains a nationally reportable disease in the United States.^††††^ These findings can help to target surveillance efforts to the most common brain and eye defects associated with Zika virus infection during pregnancy should a Zika virus outbreak reemerge, and might provide a signal to the reemergence of Zika virus, particularly in geographic regions without ongoing comprehensive Zika virus surveillance

**Summary**

**What is already known about this topic?**

Zika virus infection during pregnancy can cause serious brain and eye birth defects.

**What is added by this report?**

This study describes the frequency of individual Zika-associated birth defects from the U.S. Zika Pregnancy and Infant Registry (USZPIR). Approximately 5% of infants in USZPIR had any Zika-associated brain or eye defect. Several individual brain and eye defects were more commonly reported. One third of infants with any Zika-associated birth defect had more than one defect reported.

**What are the implications for public health practice?**

Certain brain and eye defects in infants might prompt suspicion of prenatal Zika virus infection and might provide a signal to the reemergence of Zika virus, particularly in geographic regions without ongoing comprehensive Zika virus surveillance.

## Footnotes

* Maternal laboratory evidence of confirmed or possible Zika virus infection was defined as 1) Zika virus infection detected by a Zika virus RNA nucleic acid amplification test (NAAT) (e.g., reverse transcription–polymerase chain reaction [RT-PCR]) on any maternal, placental, fetal, or infant specimen (referred to as positive Zika virus NAAT) or 2) detection of recent Zika virus infection or recent unspecified flavivirus infection by serologic tests on a maternal, fetal, or infant specimen (i.e., either positive or equivocal Zika virus immunoglobulin M [IgM] and Zika virus plaque reduction neutralization test [PRNT] titer ≥10, regardless of dengue virus PRNT value; or negative Zika virus IgM, and positive or equivocal dengue virus IgM, and Zika virus PRNT titer ≥10, regardless of dengue virus PRNT titer). Infants with positive or equivocal Zika virus IgM are included, provided a confirmatory PRNT has been performed on a maternal or infant specimen. The use of PRNT for confirmation of Zika virus infection, including during pregnancy, in women and infants, is not routinely recommended in Puerto Rico; dengue virus is endemic and cross-reactivity is likely to occur in most cases (https://www.cdc.gov/zika/laboratories/lab-guidance.html). In Puerto Rico, detection of a positive Zika virus IgM result in a pregnant woman, fetus, or infant (within 48 hours after delivery) was considered sufficient to indicate possible Zika virus infection.

† U.S. territories in USZPIR are American Samoa, Puerto Rico, and the U.S. Virgin Islands; freely associated states are Federated States of Micronesia and the Marshall Islands.

§ Infants and children in Puerto Rico and the U.S. Virgin Islands are followed through age 5 years; infants and children in U.S. states and DC, and U.S. territories and freely associated states are followed through age 3 years.

¶
https://www.researchsquare.com/article/rs-1189991/v1

** Includes maternal, placental, fetal, or infant laboratory evidence of Zika virus infection based on the presence of Zika virus RNA by a positive NAAT (e.g., RT-PCR).

†† Pregnancy losses include spontaneous abortions, terminations, stillbirths, and pregnancy losses not specified. Information from prenatal or postnatal imaging and autopsy were used to determine presence of Zika-associated birth defects, although pregnancy losses often had less information reported and frequently lacked postnatal imaging that could verify prenatal findings and might identify additional abnormalities.

§§ 45 C.F.R. part 46, 21 C.F.R. part 56; 42 U.S.C. Sect. 241(d); 5 U.S.C. Sect. 552a; 44 U.S.C. Sect. 3501 et seq.

¶¶ Signs and symptoms included fever, arthralgia, conjunctivitis, rash, and other clinical signs or symptoms that are consistent with Zika virus disease.

*** Zika virus infections that occurred during the periconceptual period, which is defined as 4 weeks before last menstrual period, are included in the first trimester.

†††
https://www.cdc.gov/zika/reporting/index.html

§§§
https://wwwnc.cdc.gov/travel/page/zika-travel-information

¶¶¶ Because of the decline in the global incidence of Zika virus, testing is not currently recommended for asymptomatic pregnant persons with possible exposure (https://www.cdc.gov/zika/symptoms/diagnosis.html). However, during the 2015–2017 Zika virus outbreak in the Americas, widespread transmission and unknown impacts of Zika virus infection during pregnancy prompted CDC to recommend testing for potentially at-risk asymptomatic pregnant persons.

****
https://www.researchsquare.com/article/rs-1189990/v1

††††
https://ndc.services.cdc.gov/case-definitions/zika-virus-disease-and-zika-virus-infection-2016-06-01/
